# Heat stress risk assessment of farmers working in a hot environment: What about in Zambia?

**DOI:** 10.1016/j.joclim.2025.100457

**Published:** 2025-07-23

**Authors:** Anayawa Nyambe, Edwell S Mwaanga, Allan Mayaba Mwiinde, Charles Michelo

**Affiliations:** a Strategic Centre for Health Systems Metrics & Evaluations (SCHEME), School of Public Health, University of Zambia, P.O. Box 50110, Lusaka, Zambia; b School of Public Health, Department of Epidemiology and Biostatistics, University of Zambia, P.O. Box 50110, Lusaka, Zambia; c Department of Public Health, Mazabuka Municipal Council, Mazabuka, Zambia; d School of Veterinary Medicine, Department of Biomedical Sciences, University of Zambia, Lusaka, Zambia; e Global Health Institute, Nkwazi Research University, Mumbuluma House, PO Box 50650, Lusaka, Zambia; f Beacon Ecology, Planetary Sciences Institute, Nkwazi Research University, PO Box 50650, Lusaka, Zambia

**Keywords:** Occupational health, Farmers, Wet bulb globe temperature, Heat stress, Heat Related Illnesses, Africa

## Abstract

**Introduction:**

Farmers are vulnerable to heat stress due to the nature of their work and the environment. This cross-sectional study aimed to assess the heat stress risk among rural farmers in the Monze and Sioma districts of Zambia.

**Method:**

Wet Bulb Globe Temperature (WBGT) readings were taken by trained farmers to assess environmental heat stress risk. Additionally, structured questionnaires on heat stress risk at the workplace were used to assess the personal experience of 200 farmers from September to November 2021.

**Results:**

Male (*n* = 92) and female (*n* = 108) respondents had a mean age of 45.4 years (*SD* = 13.75) with 97.5 % of respondents being crop farmers. WBGT readings of greater than 28 °C, which is considered an upper heat stress risk threshold for a heavy workload, were recorded. Results revealed 62.5 % of the farmers were at risk of developing heat stress, with multiple factors emerging as potential contributors to the risk.

**Conclusions:**

There is a growing heat stress risk among farmers in Monze and Sioma, along with a need to investigate heat stress effects on productivity and specific drivers of heat stress that consider differential geographical and population demographic variations. Climate adaptive and mitigative measures, including Indigenous knowledge-based approaches used by communities in rural area's should be assessed.

## Highlights

• Farmers are vulnerable to heat stress as a direct health effect of climate change.
• Heat stress risk assessment and early warning systems are beneficial for farmers.
• Tree planting benefits not only the environment but also the health of farmers.
• Health and meteorological data are essential for monitoring climate change effects.

## 1 Introduction

Heat stress occurs when excess heat raises the core body temperature above 37 °C sufficient to impair normal physiological functioning [1,2]. It results in a series of adverse health conditions or heat-related illnesses such as heat cramps, heat exhaustion, heat rash and heat stroke. According to the Intergovernmental Panel on Climate Change, over the last two decades rising temperatures and heatwaves have increased mortality and morbidity worldwide [3]. Nevertheless, heat stress risk can be decreased using climate adaptive measures such as rescheduling activities to reduce heat exposure, staying in the shade, and drinking water [4]. Adaptation to heat risk, heat-related mortality, and reduced outdoor work capacities are particularly relevant for regions with warm climates [3].

In general, studies have estimated that there is an approximate 7 % increase in productivity when a workplace is maintained between 20 °C to 24 °C [5], while temperatures above 24 °C to 26 °C are associated with reduced labor productivity [1]. Consequently, potential hours of work lost due to heat have increased significantly over the past two decades [3]. In 1995, an estimated 1.4 % of total working hours valued financially at US$280 billion were lost due to heat stress worldwide [1]. This is projected to reach 2.2 % of total working hours in 2030, representing a productivity loss equivalent to 80 million full-time jobs valued at US$2400 billion [1]. Within this total, the agricultural sector accounted for 83 % of global productivity loss in 1995, and in 2030 it is projected to account for 60 % global productivity loss [1]. Thus, heat stress may pose a significant threat to occupational health, particularly for outdoor workers exposed to temperatures that cannot be easily controlled.

The agricultural sector employs over a billion people in developing nations [6]. Farmers are at high risk as they work under high pressure for extended hours in heat and high humidity, suffer from dehydration, and often lack sufficient knowledge regarding prevention from heat exposure [5]. Their heat stress risk level is influenced by the environmental factors of high temperature, high humidity, radiant heat from the sun, and minimal wind speed [1,7].

As a result of extreme temperatures, tropical and subtropical countries with higher rates of working poverty, informal employment, and subsistence agriculture are at high risk of heat stress [1]. Unfortunately, assessments on the effects of climate change on health have been limited in low-income countries. In Nigeria, it was found that farmers frequently experience heat exhaustion [8]; in Ghana, farmers face heat stress risk in living spaces as well as during outdoor work [9]; in Gambia, fetal strain and possible adverse birth outcomes were attributed to heat stress and heat strain exposure among pregnant farmers [10]. In Zambia, heat stress studies have focused on the mining sector where a heat risk assessment found that miners exposed to temperatures far above the occupational exposure limit of 31 °C were at risk of developing heat rashes and heat stroke [11]. Studies on Zambian farmers and climate change have focused on adaptation [12], perceptions [13,14], agricultural practices [15], and economic impacts [16,17] without any mention of occupational health.

A previous climate assessment found that Zambia’s temperatures had warmed by 1.3 °C at an average rate of 0.29 °C per decade from 1960 to 2003 [18]. These temperatures are projected to increase by 1.9 °C and 2.3 °C by 2050 and 2100 respectively in a Representative Concentration Pathway (RCP) 4.5 scenario and by 3.1 °C to 4.7 °C by 2050 and 2100 respectively in an RCP 8.5 scenario [15]. Therefore, this study aimed to assess the heat stress risk among rural farmers in two different ecological zones of Zambia. It is hypothesized that farmers are at risk of developing heat stress due to farming practices, environmental factors, physiological experiences and poor adaptive practices.

## 2 Materials and methods

### 2.1 Study design and population

A cross-sectional pilot study was carried out in the Monze and Sioma districts from September to November 2021. As the objective of the study was to assess heat stress risk among rural farmers, Wet Bulb Globe Temperature (WBGT) readings were taken using measurement devices to quantify environmental heat stress, and a validated quantitative heat stress risk at the workplace (HSRW) questionnaire was piloted by research assistants to survey farmers’ perceptions and experiences while working outdoors. Concomitantly, retrospective health data were obtained from the Ministry of Health to assess trends regarding heat-related illnesses in Zambia, to illustrate the potential historical documentation of heat-related illness. Triangulation of data from these different methods was undertaken to provide an overview of the environment and possible health risks to farmers [19].

A sample of 200 farmers (Monze: *n* = 100, Sioma: *n* = 100) was used to pilot the HSRW tool in Monze and Sioma, exceeding sample size guidelines recommended for comparable pilot studies assessing feasibility [20]. The participants were required to be male or female small-scale rural farmers at least 18 years old residing in the selected districts. The identities of the participants and the information they provided were treated as confidential. Ethical approval for conducting this study was obtained from Eres Converge (Lusaka, Zambia).

### 2.2 Study instruments

The HSRW questionnaire was used to collect data from farmers. The questionnaire was divided into two parts. Social demographics (gender, age, education, employment, income, religion, marital status, living situation, household headship) and farming practices (farming systems, years of experience, farm labor) were assessed with questions developed by the author and adapted from existing tools [21,22]. The validated HSRW questionnaire developed by Dehghan et al. [23] with questions on the environment (air temperature, humidity, air flow), farming/work practices (object surface temperature, physical intensity, body posture), physiological experiences (sweating, fatigue, thirst, heat intensity, symptoms), and adaptative practices (type of clothing, color of clothing, Personal Protective Equipment [PPE]), was used to assess heat stress. See Supplementary Material 1 for the study tool.

### 2.3 Study participant sampling design

Monze and Sioma were purposely selected due to their cultural and ecological differences. Monze is primarily occupied by the Tonga tribe and is located in Southern Province in the Agro-Ecological Zone (AEZ) II which is characterized by high altitude of 900 m to 1300 m above sea level, having 800 mm to 1000 mm mean annual rainfall, and 21.2 °C mean annual temperature ranging from 6.3 °C to 33.7 °C [24]. Sioma is primarily occupied by the Lozi tribe and is located in the Western Province in the AEZ I with low altitude 300 m to 900 m above sea level, having less than 800 mm mean annual rainfall, and 24.2 °C mean annual temperature which ranges from 10.3 °C to 36.5 °C [24].

The districts were stratified into north, south, west, east and central regions according to the district agricultural zoning. A maximum of two Agricultural Camps in each region were purposively sampled resulting in 10 participating camps per district. Trained research assistants fluent in the local language were responsible for data collection from the study participants in villages in the selected camps. A maximum of 10 farmers were conveniently sampled per camp.

### 2.4 Weather data

Heat Stress WBGT Meters (Model HT30, Extech® Instruments, Vermont, USA) were used to assess outdoor heat stress risk among farmers. Since existing weather stations occasionally have missing data and were not available in the selected study districts, the heat stress devices provided more accurate measurements. WBGT (unit: °C) was specifically designed as a measure for heat stress risk for work activity assessments [1]. It accounts for temperature (Dry Bulb Temperature), humidity (Wet Bulb Temperature) and solar radiation (Globe Temperature) [25].

In each region of the Monze and Sioma districts, farmers volunteered and were trained to record WBGT readings from 06:00 to 18:00 hours as they did their daily activities. The hourly readings were taken from September 1 to November 30, 2021. This time period was chosen because these are among the hottest months of the year in Zambia when farmers may prepare fields before the rainy season, with October having the highest average temperature of 31.8 °C (27.7 °C to 36.5 °C) [24].

### 2.5 National health data

In order to assess whether heat-related illnesses have been occurring in the country, national data from the Ministry of Health was collected to observe trends. Heat illness occurs due to an increase in body temperature [26]. However, the only true heat-related illness with data available from the Ministry of Health was heat rash for the time period from 2011 to 2020 (cumulative total of 31,419 cases). Due to limited data in the provided data sets, only total cases per year were tabulated without aggregation by gender and age.

### 2.6 Data analysis

The regional WBGT readings were entered into an Excel spreadsheet (Microsoft, Redmond, WA, USA), converted into district weekly averages and graphed. Using the Statistical Package for the Social Sciences (IBM SPSS23 software for Windows, Microsoft), standard deviation, mean minimum and mean maximum WBGT readings, and coefficient of variation of all daily measurements for each month were recorded. WBGT readings were used as an objective measure of overall risk of heat stress based on prior studies of heat stress risk and WBGT.

The HSRW questionnaire data was entered into Excel and then transferred to SPSS23 where data were cleaned. A number of statistical analyses were conducted to determine social demographic characteristics, overall HSRW questionnaire scores, and significant predictors of heat stress risk.

The HSRW questionnaire score was calculated using the Deghan et al.. calculation sheet, where each question is given a primary score (non-responses were given a value of zero) and multiplied by a coefficient [23]. The sum of these becomes the overall score. A score of less than 13.5 indicates that a participant is at safe level and has no or low risk; a score between 13.6 to 18 indicates an alarming level where potential exists for heat-induced illnesses to occur and more precise evaluation of heat stress is needed; and a score greater than 18 is considered a dangerous level indicating that the onset of heat-induced illness is very likely and appropriate control measures should be taken as soon as possible to reduce risk [23].

Descriptive statistics of categorical data were reported in frequencies and percentages. A chi-square test was used to determine the association between variables while a binary logistic regression analysis was used to identify predictors of heat stress, with odds ratio as the measure of effect. The significance level was set at 0.05.

Yearly cases of heat related illnesses were tabulated in MS-Excel and graphed to observe trends.

## 3 Results

### 3.1 Sociodemographic characteristics and practices

Male (*n* = 92) and female (*n* = 108) farmers were aged between 19 and 89 years (*M* = 45.4, *SD* = 13.75). The majority had not completed secondary school (*n* = 158, 79.0 %), were married (*n* = 141, 71.9 %), and living with their biological children (*n* = 146, 73.0 %). The majority (97.5 %, *n* = 195) grew crops and had practiced farming for an average of 20.9 years (*SD* = 14.73). Table 1 summarizes the demographics of the participants.

**Table 1:** Sociodemographic characteristics and farming practices of the study participants.

		Total (*N* = 200)	Monze (*N* = 100)		Sioma (*N* = 100)	
		*N* (%)	Female *N* (%)	Male *N* (%)	Female *N* (%)	Male *N* (%)
Age	24 years and below	13 (6.5)	2 (3.9)	2 (4.1)	6 (10.5)	3 (7.0)
	25 – 29 years	10 (5.0)	3 (5.9)	4 (8.2)	2 (3.5)	1 (2.3)
	30 – 34 years	24 (12.1)	6 (11.8)	1 (2.0)	12 (21.1)	5 (11.6)
	35 – 39 years	23 (11.6)	6 (11.8)	8 (16.3)	7 (12.3)	2 (4.7)
	40 – 44 years	30 (15.1)	8 (15.7)	5 (10.2)	8 (14.0)	9 (20.9)
	45 – 49 years	29 (14.6)	2 (3.9)	13 (26.5)	6 (10.5)	8 (18.6)
	50 – 54 years	20 (10.1)	9 (17.6)	3 (6.1)	5 (8.8)	3 (7.0)
	55 – 59 years	21 (10.6)	5 (9.8)	8 (16.3)	6 (10.5)	2 (4.7)
	60 years and above	29 (14.6)	10 (19.6)	5 (10.2)	4 (7.0)	10 (23.3)
	*Total N (%)*	199 (99.5)	51 (100.0)	49 (100.0)	56 (98.2)	43 (100.0)
Education level	No formal education	7 (3.5)	1 (2.0)	0 (0.0)	3 (5.3)	3 (7.0)
	Incomplete primary school	32 (16.0)	8 (15.7)	5 (10.2)	13 (22.8)	6 (14.0)
	Complete primary school	47 (23.5)	12 (23.5)	8 (16.3)	19 (33.3)	8 (18.6)
	Incomplete secondary school	72 (36.0)	21 (41.2)	28 (57.1)	11 (19.3)	12 (27.9)
	Complete secondary school	31 (15.5)	8 (15.7)	5 (10.2)	10 (17.5)	8 (18.6)
	Incomplete tertiary education, without diploma/degree	2 (1.0)	0 (0.0)	1 (2.0)	0 (0.0)	1 (2.3)
	Complete tertiary education, with diploma/degree	9 (4.5)	1 (2.0)	2 (4.1)	1 (1.8)	5 (11.6)
	*Total N (%)*	200 (100.0)	51 (100.0)	49 (100.0)	57 (100.0)	43 (100.0)
Income	Save money	84 (42.0)	24 (47.1)	18 (36.7)	21 (36.8)	21 (48.8)
	Just get by (can afford basic needs)	133 (66.5)	34 (66.7)	30 (61.2)	40 (70.2)	29 (67.4)
	Spent some savings (and borrowed money)	60 (30.0)	9 (17.7)	13 (26.5)	20 (35.1)	18 (41.9)
	*Total N ( %)*	200 (100.0)	51 (100.0)	49 (100.0)	57 (100.0)	43 (100.0)
Religion	Catholic	49 (25.0)	12 (23.5)	9 (18.4)	16 (28.1)	12 (27.9)
	Christian Protestant	143 (72.9)	38 (74.5)	37 (75.5)	39 (68.4)	29 (67.4)
	Other	4 (2.0)	0 (0.0)	0 (0.0)	2 (3.5)	2 (4.7)
	*Total N (%)*	196 (98.0)	50 (98.0)	46 (93.9)	57 (100.0)	43 (100.0)
Marital Status	Single never married	35 (17.8)	10 (19.6)	2 (4.1)	19 (33.3)	4 (9.3)
	Married	141 (71.9)	28 (54.9)	43 (87.8)	32 (56.1)	38 (88.4)
	Widowed	15 (7.6)	10 (19.6)	0 (0.0)	5 (8.8)	0 (0.0)
	Divorced/separated	5 (2.5)	2 (3.9)	1 (2.0)	1 (1.8)	1 (2.3)
	*Total N (%)*	196 (98.0)	50 (98.0)	46 (93.9)	57 (100.0)	43 (100.0)
Living Situation	Household head	132 (66.0)	18 (35.3)	42 (85.7)	29 (50.9)	43 (100.0)
	Living alone	2 (1.0)	1 (2.0)	0 (0.0)	0 (0.0)	1 (2.3)
	Living with a partner	117 (58.5)	26 (51.0)	33 (67.3)	28 (49.1)	30 (69.8)
	Living with biological children	146 (73.0)	36 (70.6)	34 (69.4)	45 (78.9)	31 (72.1)
	Living with step-children	39 (19.5)	7 (13.7)	9 (18.4)	14 (24.6)	9 (20.9)
	Living with other family members	85 (42.5)	31 (60.8)	25 (51.0)	15 (26.3)	14 (32.6)
	*Total N (%)*	200 (100.0)	51 (100.0)	49 (100.0)	57 (100.0)	43 (100.0)
Farming Practices	Crop Farming	195 (97.5)	50 (98.0)	48 (98.0)	55 (96.5)	42 (97.7)
	Livestock Farming	141 (70.5)	42 (82.4)	41 (83.7)	28 (49.1)	30 (69.8)
	Fruit Trees Farming	59 (29.5)	0 (0.0)	5 (10.2)	28 (49.1)	26 (60.5)
	Fish Farming	14 (7.0)	0 (0.0)	0 (0.0)	3 (5.3)	11 (25.6)
	Poultry Farming	137 (68.5)	36 (70.6)	43 (87.8)	33 (57.9)	25 (58.1)
	*Total N (%)*	200 (100.0)	51 (100.0)	49 (100.0)	57 (100.0)	43 (100.0)
Years of farming	9 years and below	43 (21.7)	10 (19.6)	12 (24.5)	13 (22.8)	8 (18.6)
	10 – 19 years	56 (28.2)	17 (33.3)	13 (26.5)	17 (29.8)	9 (20.9)
	20 – 29 years	48 (24.2)	12 (23.5)	14 (28.6)	11 (19.3)	11 (25.6)
	30 – 39 years	23 (11.6)	5 (9.8)	5 (10.2)	7 (12.3)	6 (14.0)
	40 – 49 years	18 (9.0)	3 (5.9)	2 (4.1)	8 (14.0)	5 (11.6)
	50 years and above	10 (5.0)	3 (5.9)	2 (4.1)	1 (1.8)	4 (9.3)
	*Total N (%)*	198 (99.0)	50 (98.0)	48 (98.0)	57 (100.0)	43(100.0)

### 3.2 WBGT readings

**Fig. 1:**
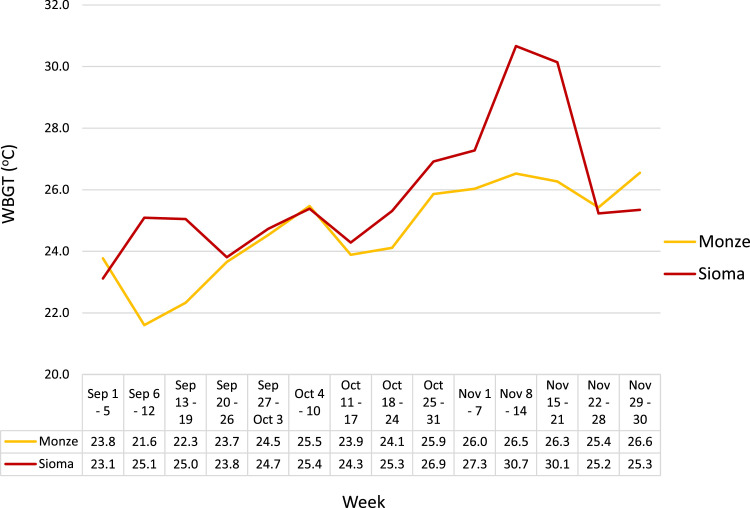
Average weekly heat stress WBGT readings in Monze and Sioma from September to November 2021. Note that in Monze, the lowest daily average reading of 19.4 °C was recorded on September 11, 2021, while the highest readings of 27.3 °C were on November 9 and 14, 2021. In Sioma, the lowest daily average reading of 20.8 °C was recorded on September 1, 2021 and the highest at 33.2 °C was recorded on November 16, 2021.

In general, the WBGT readings (Fig. 1 and Table A.1), indicated that farmers were at risk of developing heat stress due to environmental factors. Monze had lower WBGT readings (*M* = 24.6 °C, *SD* = 1.66) compared to Sioma (*M* = 26.0 °C, *SD* = 2.48), *t* = −4.282, 95 %CI = [−1.96, −0.72], *df* = 180, *p* < 0.001.

**Table A.1:** Summary of monthly WBGT readings in Monze and Sioma.

Month	Time period	Monze WBGT					Sioma WBGT				
		*M*	*SD*	Min	Max	COV	*M*	*SD*	Min	Max	COV
September (*n* = 30)	06:00	17.4	1.59	15.3	21.0	9.1 %	17.5	1.40	13.8	20.0	8.0 %
	09:00	22.8	1.63	19.2	25.2	7.2 %	24.5	1.16	22.1	27.1	4.7 %
	12:00	28.0	1.60	23.9	31.6	5.7 %	28.2	1.42	25.3	30.9	5.0 %
	15:00	26.0	1.88	20.1	29.4	7.2 %	28.1	1.84	23.5	30.6	6.6 %
	18:00	20.9	1.77	16.8	23.8	8.5 %	23.9	1.80	27.7	19.1	7.5 %
Average	06:00 - 18:00	23.0	1.36	19.4	25.0	5.9 %	24.4	1.24	20.8	26.6	5.1 %
October (*n* = 31)	06:00	20.1	1.20	17.2	22.7	5.9 %	19.7	1.84	16.0	23.3	9.3 %
	09:00	24.6	1.29	21.0	27.0	5.3 %	25.7	1.42	23.0	28.3	5.5 %
	12:00	28.8	1.47	24.4	30.9	5.1 %	29.4	1.76	24.9	32.8	6.0 %
	15:00	26.8	1.18	24.0	28.9	4.4 %	28.1	1.07	26.0	30.1	3.8 %
	18:00	23.0	1.00	20.9	24.9	4.4 %	23.9	1.13	21.2	25.8	4.7 %
Average	06:00 - 18:00	24.7	1.04	22.1	26.3	4.2 %	25.4	1.15	23.1	27.9	4.5 %
November (*n* = 30)	06:00	22.7	0.71	20.8	24.0	3.1 %	25.2	4.76	19.5	33.7	18.9 %
	09:00	26.4	0.91	24.0	27.9	3.4 %	28.6	3.59	23.1	36.1	12.6 %
	12:00	29.7	1.30	26.6	32.5	4.4 %	31.8	2.75	27.4	37.0	8.7 %
	15:00	27.8	1.10	25.6	30.1	3.9 %	29.2	2.45	24.8	34.6	8.4 %
	18:00	23.9	0.70	22.4	25.1	2.9 %	25.6	2.66	21.1	31.3	10.4 %
Average	06:00 - 18:00	26.1	0.73	24.2	27.3	2.8 %	28.1	2.87	23.7	33.2	10.2 %

### 3.3 Heat stress risk at the workplace questionnaire assessment

Table 2 highlights the total scores obtained by the farmers from the HSRW questionnaire. The heat stress risk scores (Table 2) were influenced by the significant variables in Tables 3 and A.2.

**Table 2:** Total heat stress risk at the workplace questionnaire scores.

Heat Stress Risk Assessment	Total N ( %)	Monze (*n* = 100)		Sioma (*n* = 100)	
		Male (*n* = 49)	Female (*n* = 51)	Male (*n* = 43)	Female (*n* = 57)
Safe level (score: ≤ 13.5)	75 (37.5)	19 (38.8)	24 (47.1)	15 (34.9)	17 (29.8)
Alarm level (score: 13.6 – 18.0)	55 (27.5)	17 (34.7)	10 (19.6)	14 (32.6)	14 (24.6)
Danger level (score: ≥18.0)	70 (35.0)	13 (26.5)	17 (33.3)	14 (32.6)	26 (45.6)

**Table 3:** Farming practices, environmental factors, physiological experiences and adaptative factors linked to heat stress risk among rural farmers.

Heat Stress Risk				Chi-square test				
Monze		Safe Level	Risk Level	OR	95 % CI	χ^2^	*df*	*p*
Level of mobility while working^1^	High Low	18 25	49 8	0.1	[0.05, 0.31]	21.6	1	<0.001
Experiencing dizziness	Yes No	4 39	27 30	0.1	[0.04, 0.36]	16.6	1	<0.001
Experiencing muscle pain	Yes No	6 37	19 38	0.3	[0.12, 0.90]	4.9	1	0.03


Sioma		Safe Level	Risk Level	OR	95 % CI	χ^2^	*df*	*p*
Practicing fruit tree farming	Yes No	10 22	44 24	0.2	[0.10, 0.61]	9.8	1	0.002
Practicing poultry farming	Yes No	14 18	44 24	0.4	[0.18, 1.00]	3.9	1	0.05
Using a shield during work*	Yes No	4 28	0 68	21.6	[1.13, 415.06]	8.9	1	0.003
Using gloves during work*	Yes No	5 27	0 68	27.4	[1.46, 512.48]	11.2	1	0.001
Using an apron during work*	Yes No	2 30	0 68	11.23	[0.52, 241.00]	4.3	1	0.04
Experiencing mild headache while working	Yes No	4 28	23 45	0.3	[0.09, 0.89]	5.0	1	0.03
Experiencing lower concentration while working	Yes No	7 25	5 63	3.5	[1.02, 12.16]	4.3	1	0.04
Experiencing rash while working*	Yes No	0 32	13 55	0.1	[0.00, 1.10]	7.0	1	0.01

⁎ OR and 95 %CI adjusted for 0 cell count reading by adding a 0.5 Haldane-Anscombe correction. Note that all OR were calculated as raw 2 × 2 contingency tables.

1 Level of mobility while working, responses: High includes Standing with a high mobility, and Usually I am walking; Low includes Usually sitting, and Usually standing with low mobility.

**Table A.2:** Indicators of heat stress risk among farmers in Monze and Sioma.

Monze	Heat Stress		Sioma	Heat Stress	
Air Temperature1*	Safe	Risk	Air Temperature^1^	Safe	Risk
Warm (*n* = 80)	23	57	Warm (*n* = 85)	21	64
Cool (*n* = 20)	20	0	Cool (*n* = 15)	11	4
χ^2^ = 33.1, OR = 0.0, 95 % CI = [0.00, 0.17], *df* = 1, *p* < 0.05			χ^2^ = 13.9, OR = 0.1, 95 % CI = [0.03, 0.42], *df* = 1, *p* < 0.05		
**Air Flow**2⁎	**Safe**	**Risk**	**Air Flow**^2^	**Safe**	**Risk**
Warm (*n* = 84)	27	57	Warm (*n* = 75)	16	59
Cold (*n* = 16)	16	0	Cold (*n* = 25)	16	9
χ^2^ = 25.2, OR = 0.0, 95 % CI = [0.00, 0.25], *df* = 1, *p* < 0.05			χ^2^ = 15.7, OR = 0.2, 95 % CI = [0.06, 0.41], *df* = 1, *p* < 0.05		
**Humidity**^3^	**Safe**	**Risk**	**Humidity**^3^	**Safe**	**Risk**
Severe (*n* = 50)	11	39	Severe (*n* = 89)	22	67
Minimal (*n* = 50)	32	18	Minimal (*n* = 11)	10	1
χ^2^ = 18.0, OR = 0.2, 95 % CI = [0.07, 0.38], *df* = 1, *p* < 0.05			χ^2^ = 19.7, OR = 0.0, 95 % CI = [0.00, 0.27], *df* = 1, *p* < 0.05		
**Heat Intensity**^4^	**Safe**	**Risk**	**Heat Intensity**^4^	**Safe**	**Risk**
Severe (*n* = 68)	24	44	Severe (*n* = 49)	5	44
Minimal (*n* = 32)	19	13	Minimal (*n* = 51)	27	24
χ^2^ = 5.1, OR = 0.4, 95 % CI = [0.16, 0.89], *df* = 1, *p* < 0.05			χ^2^ = 21.0, OR = 0.1, 95 % CI = [0.03, 0.30], *df* = 1, *p* < 0.05		
**Surface Temperature**^5^	**Safe**	**Risk**	**Surface Temperature**^5^	**Safe**	**Risk**
Hot (*n* = 83)	29	57	Hot (*n* = 38)	7	31
Cold (*n* = 17)	14	3	Cold (*n* = 62)	25	37
χ^2^ = 12.9, OR = 0.1, 95 % CI = [0.03, 0.41], *df* = 1, *p* < 0.05			χ^2^ = 5.2, OR = 0.3, 95 % CI = [0.13, 0.88], *df* = 1, *p* < 0.05		
**Color of Clothing**	**Safe**	**Risk**	**Color of Clothing**	**Safe**	**Risk**
Dark (*n* = 35)	21	14	Dark (*n* = 82)	21	61
Light (*n* = 65)	22	43	Light (*n* = 18)	11	7
χ^2^ = 6.3, OR = 2.9, 95 % CI = [1.25, 6.85], *df* = 1, *p* < 0.05			χ^2^ = 8.5, OR = 0.2, 95 % CI = [0.08, 0.64], *df* = 1, *p* < 0.05		
**Wearing PPE**	**Safe**	**Risk**	**Wearing PPE**	**Safe**	**Risk**
Yes (*n* = 25)	6	19	Yes (*n* = 21)	14	7
No (*n* = 75)	37	38	No (*n* = 79)	18	61
χ^2^ = 4.9, OR = 0.3, 95 % CI = [0.12, 0.90], *df* = 1, *p* < 0.05			χ^2^ = 14.7, OR = 6.8, 95 % CI = [2.38, 19.34], *df* = 1, *p* < 0.05		
Wearing a Mask*	**Safe**	**Risk**	Wearing a Mask*	**Safe**	**Risk**
Yes (*n* = 9)	0	9	Yes (*n* = 4)	4	0
No (*n* = 91)	43	48	No (*n* = 96)	28	68
χ^2^ = 7.5, OR = 0.1, 95 % CI = [0.00, 1.04], *df* = 1, *p* < 0.05			χ^2^ = 8.9, OR = 21.6, 95 % CI = [1.13, 415.06], *df* = 1, *p* < 0.05		
**Wearing a Hat**	**Safe**	**Risk**	**Wearing a Hat**	**Safe**	**Risk**
Yes (*n* = 13)	1	12	Yes (*n* = 8)	6	2
No (*n* = 87)	42	45	No (*n* = 92)	26	66
χ^2^ = 7.6, OR = 0.1, 95 % CI = [0.01, 0.72], *df* = 1, *p* < 0.05			χ^2^ = 7.4, OR = 7.6, 95 % CI = [1.44, 40.19], *df* = 1, *p* < 0.05		
**Thirst**6*	**Safe**	**Risk**	**Thirst**^6^	**Safe**	**Risk**
Severe (*n* = 95)	38	57	Severe (*n* = 92)	25	67
Minimal (*n* = 5)	5	0	Minimal (*n* = 8)	7	1
χ^2^ = 7.0, OR = 0.1, 95 % CI = [0.00, 1.13], *df* = 1, *p* < 0.05			χ^2^ = 12.3, OR = 0.1, 95 % CI = [0.01, 0.46], *df* = 1, *p* < 0.05		
**Sweating**^7^	**Safe**	**Risk**	**Sweating**^7^	**Safe**	**Risk**
Severe (*n* = 66)	11	55	Severe (*n* = 76)	11	65
Minimal (*n* = 34)	32	2	Minimal (*n* = 24)	21	3
χ^2^ = 54.9, OR = 0.0, 95 % CI = [0.00, 0.06], *df* = 1, *p* < 0.05			χ^2^ = 44.7, OR = 0.0, 95 % CI = [0.01, 0.10], *df* = 1, *p* < 0.05		
**Fatigue**^8^	**Safe**	**Risk**	**Fatigue**^8^	**Safe**	**Risk**
Severe (*n* = 53)	12	41	Severe (*n* = 58)	6	52
Minimal (*n* = 47)	31	16	Minimal (*n* = 42)	26	16
χ^2^ = 19.1, OR = 0.2, 95 % CI = [0.06, 0.37], *df* = 1, *p* < 0.05			χ^2^ = 29.8, OR = 0.1, 95 % CI = [0.03, 0.20], *df* = 1, *p* < 0.05		

⁎ OR and 95 %CI adjusted for 0 cell count reading by adding a 0.5 Haldane-Anscombe correction. Note that all OR were calculated as raw 2 × 2 contingency tables.

1 Air Temperature responses: Warm includes Slightly warm, Warm, Very Warm; Cool includes Cold, Slightly Cool, Normal.

2 Air Flow responses: Warm includes Sense of stability in the gentle flow of air or warm air, The moderate flow of warm air, and Extreme current of hot weather; Cool includes The existence of cold weather circulation, The existence of cold weather current, and Gentle stream of pleasing air.

3 Humidity level responses: Severe includes Clothes sticking to the skin surface, Fully wet skin, Sweat loss from the skin surface; Minimal includes Dry (a feeling of dryness in the mouth and throat), Appropriate and desirable, and Wet skin.

4 Heat Intensity responses: Severe includes I’m annoyed, I’m very annoyed, and I’m so annoyed that I want to quit my job posts; Minimal includes I’m not annoyed, and I’m a little annoyed.

5 Surface Temperature responses: Hot includes I feel hot, Their heat cannot be tolerable, and If my skin is in touch with them I will be burnt; Cold includes I feel too cold, I feel cold, I feel cool and I do not feel cold or hot.

6 Thirst responses: Severe includes I get thirsty, I get very thirsty, and I get so thirsty that my mouth and throat get dry and they can’t be wet with saliva; Minimal includes I don’t get thirsty, and I get a little thirsty.

7 Sweating responses: Severe includes So severe underwear clothing gets wet, So severe it is felt on face, So severe it flows all over body; Minimal includes I do not feel like sweating, On the armpit and inguinal, and On the chest and back.

8 Fatigue responses: Severe includes Exhausted, and So exhausted a break is desired; Minimal includes Not tired at all, A little tired, and Tired.

In Table 3, Table A.2, farmers were considered to be either at a safe level or at-risk level (combined alarm and danger) of developing heat stress as determined by the HSRW questionnaire scores. According to Table 3, experiencing symptoms such as dizziness, muscle pain, mild headache, and rash, as well as certain farming practices (poultry and fruit tree cultivation) increased the risk of developing heat stress. Protective factors may include reducing mobility and wearing PPE (shields, gloves and apron). Table A.2 in the Appendix summarizes working conditions and symptom factors associated with increased risk of heat stress (air temperature, air flow, heat intensity, surface temperature, thirst, sweating, fatigue) that affected both districts. However, certain factors, such as humidity level, clothing color, and the use of PPE (e.g. masks or hats), offered protection, though in some cases, the effectiveness varied by district.

A binary logistic regression analysis was performed using the HSRW questionnaire scores for risk level (combined alarm and danger) and safe level, against sociodemographic characteristics and farming practices as listed in Table 1. The results using this approach indicated that farmers who did not practice fruit tree farming were significantly more likely to experience heat stress (*β* = –1.08, OR = 0.3, 95 %CI = [0.16, 0.74], Wald = 7.5, *df* = 1, *p* = 0.01). The model explained 23 % of variance (Nagelkerke R² = 0.23), with a Cox & Snell R² of 0.17, and correctly predicted 70.7 % of cases.

### 3.4 National health data

**Fig. A.1:**
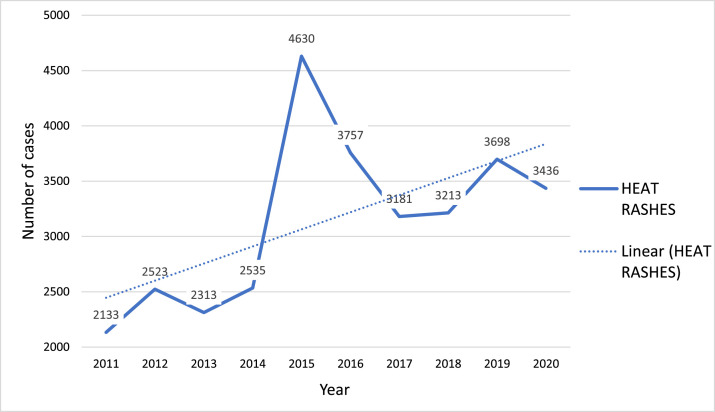
Annual heat rash cases in Zambia, 2011 - 2020, accessed from the Ministry of Health data base.

There was an increasing trend of national heat rash cases which peaked in 2015 (*n* = 4630). For graphic representation see Fig. A.1 in the appendix.

## 4 Discussion

In this study, heat stress risk was assessed by taking WBGT measurements, surveying farmers with a standard questionnaire, and assessing historical health data. Both measured values (Fig. 1 and Table A.1) and personal assessment (Table 2, Table 3, Table A.2) show that farmers are at risk of facing heat stress.

The WBGT readings were higher in Sioma than in Monze as expected, since Sioma is located in a hotter region [24]. This is further impacted by the fact that agriculture is a heavy workload activity [27], and previous studies have determined heat stress thresholds to be from 25 °C to 28 °C for a heavy 30 min workload with 15 min rest cycles [28]. According to Table A.1 in the Appendix, monthly averages fell within this threshold, though some readings exceeded it, indicating a need for cost-effective climate adaptation practices. However, rural farmers may have inadequate coping strategies to reduce heat stress risk [29]. Therefore, we suggest developing government-led interventions and implementing locally tailored heat-health response plans with effective weather monitoring facilities to assist in scheduling of farming activities. This should be done alongside initiatives to improve water access and promote indigenous knowledge-based practices such as building "*insaka"*, traditional simple grass-thatched structures made from locally sourced materials which may provide shade for farmers near fields [30].

The overall HSRW questionnaire score (Table 2) determined that only 37.5 % (*n* = 75) of farmers had no or low heat strain. At-risk groups (*n* = 125, 62.5 %) included those with levels indicating the potential of developing heat-induced illnesses and those very likely to develop them requiring urgent prevention measures. Considering the high-risk population, public communication strategies may be necessary to raise heat risk awareness among farmers.

In support of the hypothesis, several variables indicated increased heat stress risk among the rural farmers (Table 3, Table A.2). Indictors arose from physiological experiences, farming practices and attire, and environmental factors that have been identified in previous studies [1,4,5,7]. PPE may increase [31] or decrease [32] heat stress risk if not properly used and may depend on additional factors such as frequency of use and material. For instance, lightweight, breathable fabrics are preferable to heavy, non-breathable materials, and consistently wearing wide-brimmed hats provide better sun protection than caps. Interestingly, reporting experiencing lower concentration signaled decreased risk, possibly due to language-related misunderstandings where participants relate lower concentration to relaxed work activities. At first glance, cultivating fruit trees appears to increase farmers' risk of heat stress. However, after accounting for other factors in the regression analysis, fruit tree farming may lower this risk by creating a cooler environment, suggesting that tree planting initiatives as implemented in Sioma have the potential to mitigate heat stress [33].

Considering the increasing trend of national heat rash cases (Fig. A.1) and the evidence of heat stress risk among farmers, including heat related illnesses among notifiable diseases may be of importance as they can be expected to increase with increasing climate change. Rising global temperatures, prolonged heatwaves, and shifting weather patterns can increase the incidence of heat-related illnesses, and ultimately reduce productivity, affecting livelihoods and food security. Unfortunately, availability and access of health and meteorological data remains a challenge due to inadequate recording systems with health data being generally limited.

## 5 Limitations

This study was limited by focusing on the Monze and Sioma districts which are not representative of the whole country. Survey sites were selected with the assistance of agricultural officers, and farmers participated voluntarily, which may have introduced selection bias. Additionally, despite being adequate for a pilot study, the sample size and three-month study period was minimal. A larger randomized sample in more locations would provide more representative data. Furthermore, heat stress was assessed using Heat Stress WBGT Meters Model HT30, with farmers trained to take and record readings. While WBGT is a standard measure, factors such as calibration, placement, and variability in data collection techniques may have affected measurement accuracy. Hence, questionnaires and historical health data were used to support the findings. Future studies could enhance data reliability by incorporating cross-validation methods using different brands of measurement devices and implementing quality control checks.

## 6 Conclusion

Heat stress is a growing risk among farmers in the Monze and Sioma Districts of Zambia. The WBGT readings indicated that farmers in these districts were at risk of developing heat stress, with key predictors including family situation and agroforestry practices. National data are inadequate to facilitate understanding of the impacts of heat on the population. Consideration should be given towards developing adaptative techniques which could help farmers, other vulnerable populations and the general community, such as localized weather stations along with early warning systems that may provide heat stress forecasting services. To combat the risk of heat stress, tree planting and other cost-effective climate adaptative, mitigative and indigenous knowledge-based practices such as providing traditional structures/shelters for shade, traditional water storage containers [30] or mist bottles, cooling vests, water breaks and altering farmers’ working hours should be considered.

## Abbreviations

AEZ Agro-Ecological Zones/Regions

HSRW Heat Stress Risk at the Workplace

PPE Personal Protective Equipment

RCP Representative Concentration Pathways

SPSS Statistical Package for the Social Sciences

WBGT Wet Bulb Globe Temperature

## Declarations

### Ethics approval and consent to participate

Ethical clearance was obtained from Eres Converge in Lusaka Zambia, reference number 2020-Aug-001. This research obtained written and informed consent from all participants. Their identities and information they provided are confidential.

Clearance for publication was obtained from the National Health Research Authority of Zambia, Ministry of Health.

## Consent to publish

Not Applicable

## Availability of data and material

The datasets during and/or analyzed during the current study are available from the author AN on reasonable request.

## Funding

The research of this article was supported by DAAD Deutshcer Akademischer Austauschdienst, German Academic Exchange Service) within the framework of the ClimapAfrica program with funds of the Federal Ministry of Education and Research. The publisher is fully responsible for the content.

## CRediT authorship contribution statement

**Anayawa Nyambe:** Writing – review & editing, Writing – original draft, Visualization, Software, Resources, Project administration, Methodology, Investigation, Funding acquisition, Formal analysis, Data curation, Conceptualization. **Edwell S Mwaanga:** Writing – review & editing, Supervision. **Allan Mayaba Mwiinde:** Writing – review & editing, Visualization, Validation, Methodology. **Charles Michelo:** Writing – review & editing, Validation, Supervision, Resources, Funding acquisition, Formal analysis, Data curation, Conceptualization.

## Declaration of competing interest

The authors declare that they have no known competing financial interests or personal relationships that could have appeared to influence the work reported in this paper.

## Appendix B Supplementary materials

Image, application 1

## References

[1] International Labour Office (2019).

[2] Kjellstrom T., Briggs D., Freyberg C., Lemke B., Otto M., Heat Hyatt O. (2016). Human performance, and occupational health: a key issue for the assessment of global Climate change impacts. Annu Rev Public Health.

[3] IPPC (2022). Contribution Of Working Group II To The Sixth Assessment Report Of The Intergovernmental Panel On Climate Change.

[4] Parkes B., Cronin J., Dessens O., Sultan B. (2019). Climate changes in Africa: cost of mitigating heat stress. Clim Change.

[5] Lundgren K.K., Kuklane K., Gao C., Holmér I. (2013). Effects of heat stress on working populations when facing climate change. Ind Health.

[6] Cheong D., Jansen M., Peters R. (2013). International Labour Office And United Nations Conference On Trade And Development. Shared Harvests: Agriculture, Trade, And Employment.

[7] Park J., Kim Y., Oh I. (2017). Factors affecting heat-related diseases in outdoor workers exposed to extreme heat. Ann Occup Env Med.

[8] Sadiq L.S., Hashim Z., Osman M. (2019). The impact of heat on health and productivity among maize farmers in a tropical climate area. J Env Public Health.

[9] Frimpong K., Van Etten E.J., Oosthuzien J., Nunfam V.F (2017). Heat exposure on farmers in northeast Ghana. Int J Biometeorol.

[10] Bonell A., Sonko B., Badjie J., Samateh T., Saidy T., Sosseh F. (2022). Environmental heat stress on maternal physiology and fetal blood flow in pregnant subsistence farmers in The Gambia, west Africa: an observational cohort study. Lancet Planet Health.

[11] Malambo L. (2014).

[12] Kajoba G.M. (2017). Building the resilience of food production systems of small scale farmers in the context of climate change In rural Zambia: the case of Kafwambila Village in Sinazongwe District, Southern Zambia. Int J Multi-Discip Res.

[13] Marcantonio R.A., Attari S.Z., Evans T.P. (2018). Farmers perceptions of conflict related to water in Zambia. Sustainability.

[14] Mulenga B.P., Wineman A., Sitko N.J. (2017). Climate trends and farmers’ perceptions of climate change in Zambia. Env Manage.

[15] Hamududu B.H., Ngoma H. (2020). Impacts of climate change on water resources availability in Zambia: implications for irrigation development. Env Dev Sustain.

[16] Dumenu W.K., Takam Tiamgne X. (2020). Social vulnerability of smallholder farmers to climate change in Zambia: the applicability of social vulnerability index. SN Appl Sci.

[17] Jain S. (2007). An empirical economic assessment of impacts of climate change on agriculture in Zambia. Policy research working paper 4291. World Bank.

[18] Aid Irish (2018). Zambia Country Climate Risk Assessment Report: Irish Aid, Resilience and Economic Inclusion Team, Policy Unit.

[19] Thurmond V. (2001). The point of triangulation. J Nurs Scholarsh.

[20] Teresi J.A., Yu X., Stewart A.L., Hays R.D. (2022). Guidelines for designing and evaluating feasibility pilot studies. Med Care.

[21] Nevitte N. (2008).

[22] Nyambe A., Kampen J.K., Baboo S.K., Van Hal G. (2019).

[23] Dehghan H., Mortzavi S.B., Jafari M.J., Maracy M.R. (2015). Development and validation of a questionnaire for preliminary assessment of heat stress at workplace. J Res Health Sci.

[24] Kasali G. (2008).

[25] Cooper E., Grundstein A., Rosen A., Miles J., Ko J., Curry P. (2017). An evaluation of portable wet bulb globe temperature monitor accuracy. J Athl Train.

[26] Miners A.L. (2010). The diagnosis and emergency care of heat related illness and sunburn in athletes: a retrospective case series. J Can Chiropr Assoc.

[27] Mathiassen A., Hollema S. (2014). What is the effect of physical activity level on food consumption, energy deficiency, and dietary diversity?. Food Nutr Bull.

[28] Brimicombe C., Lo C.H.B., Pappenberger F., Di Napoli C., Maciel P., Quintino T. (2023). Wet bulb globe temperature: indicating extreme heat risk on a global grid. GeoHealth.

[29] Frimpong K., Odonkor S.T., Kuranchie F.A., Nunfam V.F. (2020). Evaluation of heat stress impacts and adaptations: perspectives from smallholder rural farmers in Bawku East of Northern Ghana. Heliyon.

[30] Nyambe A. (2024). Exploring climate change perception and heat stress adaptation among Zambian farmers using participatory tools. Reg Env Change.

[31] Tigchelaar M., Battisti D.S., Spector J.T. (2020). Work adaptations insufficient to address growing heat risk for U.S. agricultural workers. Environ Res Lett.

[32] Debela M.B., Begosaw A.M., Deyessa N., Azage M. (2023). The burdens of occupational heat exposure-related symptoms and contributing factors among workers in sugarcane factories in Ethiopia: heat stress wet bulb globe temperature meter. Saf Health Work.

[33] Africa Development Bank (October 2023). Zambia cashew infrastructure development project (CIDP). Osan Dep.

