# Supplementary material for: Heat stress risk assessment of farmers working in a hot environment: What about in Zambia?
Source: J Clim Chang Health. 2025 Jul 23;24:100457. doi: 10.1016/j.joclim.2025.100457 (PMC12851382; doi:10.1016/j.joclim.2025.100457)
Supplement: Supplementary file 1 [file mmc1.docx]

**HEAT STRESS RISK ASSESSMENT**

Name of Interviewer:

Date:

Location:

Identification Code:

**General Instructions for interviewer**

1. This questionnaire has two sections.

SECTION A: Demograhic Information

SECTION B: Heat Stress Assessment

1. Answer questions by writing in the box or marking an “X” in the box representing the desired response. For questions where there are multiple responses, respondents can choose more than one answer. For questions without answer options, write the response in the box.
2. **DEMOGRAPHIC INFORMATION**
3. Gender

_1_ Male _2_ Female

1. What is your year of birth?

Year

1. What is the highest educational level that you have attained?

_1_ No formal education

_2_ Incomplete primary school

_3_ Complete primary school

_4_ Incomplete secondary school

_5_ Complete secondary school

_6_ Incomplete college, without diploma

_7_ Complete college, with diploma

_8_ Incomplete university-level education, without degree

_9_ University-level education, with degree

1. What is your employment status?

_1_ Full time employee

_2_ Part time employee

_3_ Self employed

_4_ Retired/pensioned

_5_ Housewife/unemployed/volunteer

_6_ Student/Intern

_7_ Other, specify:

1. During the past year, did your family: *(mark all that apply)*

_1_ Save money

_2_ Just get by

_3_ Spent some savings

_4_ Spent savings and borrowed money

1. Do you belong to a religion or religious denomination?

_1_ No (do not belong to a denomination/religion)

_2_ Catholic

_3_ Christian Protestant

_4_ Orthodox

_5_ Jew

_6_ Muslim

_7_ Hindu

_8_ Buddhist

_9_ Other, specify:

1. Current marital status

_1_ Single

_2_ Married

_3_ Widowed

_4_ Divorced/separated

1. With whom do you live? *(mark all that apply)*

_1_ You live alone

_2_ Partner (Wife/Husband)

_3_ Biological children (Your own children)

_4_ Step children (Your partner’s children that are not genetically yours)

_5_ Other family members (e.g. aunties, uncles, nieces, nephews)

_6_ Other (e.g. with friends)

9. Are you the head of household?

_1_ Yes

_2_ No

1. Which types of farming do you practice? *(mark all that apply)*

_1_ Crops

_2_ Livestock

_3_ Fruit trees

_4_ Fish

_5_ Poultry (chicken, quails, ducks, guinea fowls)

_6_ Other, specify:

1. How long have you been farming? (Months, Years)

Year(s): Month(s):

1. How many people work with you on your farm?

Male: Female: Total:

1. **HEAT STRESS ASSESSMENT**
2. How do you feel your workplace air temperature?

| Very cold | Cold | Slightly Cool | Normal | Slightly warm | Warm | Very warm |
| --- | --- | --- | --- | --- | --- | --- |
|  |  |  |  |  |  |  |

1. How do you feel the humidity level of your workplace?

Dry (a feeling of dryness in the mouth and throat)

Appropriate and desirable

Wet skin

Clothes sticking to the skin surface

Fully wet skin

Sweat loss from the skin surface

1. How do you feel the temperature of adjacent surfaces (e.g. when you touch a hoe/shovel/bucket) due to contact with your hands?

I feel too cold

I feel cold

I feel cool

I do not feel cold or hot

I feel hot

Their heat cannot be tolerable

If my skin is in touch with them I will be burnt

1. How do you feel the flow of air in your workplace?

The existence of cold weather circulation

The existence of cold weather current

Gentle stream of pleasing air

Sense of stability in the gentle flow of air or warm air

The moderate flow of warm air

Extreme current of hot weather

1.
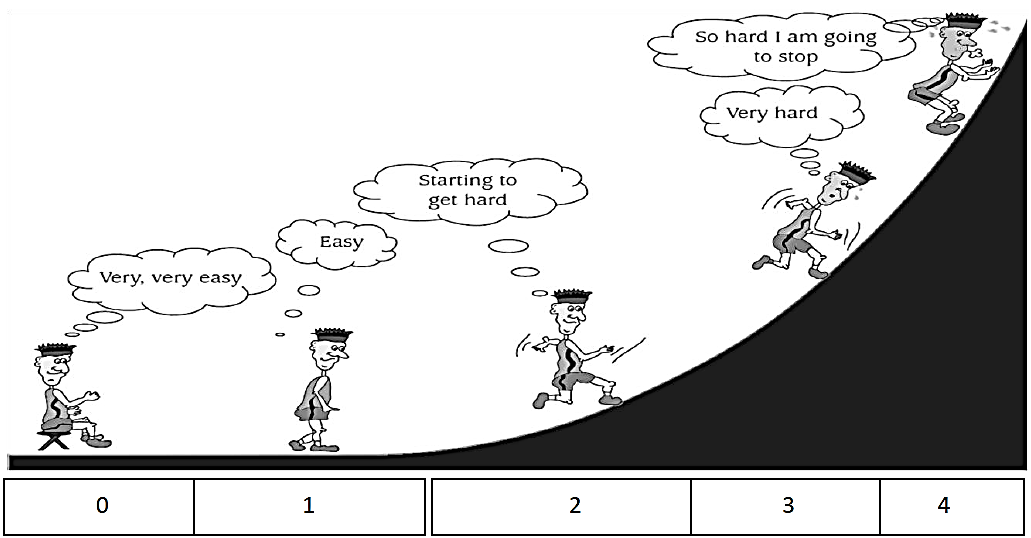
While you are working, the intensity of physical activity you do is like which of the following conditions?
2. How much is the amount of sweating throughout your working?

I do not feel like sweating

I feel the sweat on the armpit and inguinal (groin/inner thighs)

I feel the sweat on the chest and back

Sweating is so severe that the underwear clothing gets wet

Sweating is so severe that I feel it on my face

Sweating is so severe that it is flowing all over my body

1. How much fatigue you are at work?

I'm not tired at all

I'm a little tired

I'm tired

I'm exhausted

I'm so exhausted that I desire to have a break

1. How much is the intensity of your thirst when you are at work?

I don’t get thirsty

I get a little thirsty

I get thirsty

I get very thirsty

I get so thirsty that my mouth and throat get dry and they can’t be wet with saliva

1. How intensive are you suffering from heat?

I'm not annoyed

I'm a little annoyed

I'm annoyed

I'm very annoyed

I'm so annoyed that I want to quit my job posts

1. How do you feel about the size of working space within the building/work environment?

Spacious

Appropriate common space

Limited cramped space

1. How is the ventilation system in your workplace?

Active and high ventilation

Appropriate ventilation, it is not needed to be ventilated

Inadequate ventilation

Despite the lack of air conditioning, there is no ventilation

1. In which environments below you are doing your own tasks now?

Outdoors

Indoor

Both

1. What kind of clothes do you use while you work out?

T-shirts and jeans (no work clothing worm)

Normal work clothing (underwear + shirts and pants)

Full suits (underwear + work clothing coverall)

Heavy or wool clothing or winter work clothing (underwear + double cloth coveralls)

Water -proof clothing (chemical protective clothing, wind visor, leather)

Fully enclosed suit with hood and gloves

1. What color is your work clothing?

Light colors (e.g. white, cream, yellow, light blue, orange, etc.)

Dark colors (e.g. Black, dark brown, dark red and dark blue)

1. What material is your work clothing?

| Cotton | Cotton and synthetic fibers | Fireproof and water proof |
| --- | --- | --- |
|  |  |  |

1. During the work, which equipment do you use including the following personal protection equipment?

Self-contained breathing apparatus

Full- face respirator

Half-face respirator

Water proof boot

Leather apron

Anti-dust mask

Face shield

Not-cotton glove

Hat

Ear muff

None

1. What is your more often body posture when you are at work?

Usually sitting

Usually standing with low mobility

Standing with a high mobility

Usually I am Walking

1. Now which of the following symptoms do you have while you are working?

Mild headache

Dizziness

Weakness

Muscle pain

Rash

Lower concentration

None
